# The Role of Active Video-Accompanied Exercises in Improvement of the Obese State in Children: A Prospective Study from Turkey

**DOI:** 10.4274/jcrpe.2284

**Published:** 2016-09-01

**Authors:** Fatma Duman, Mehmet Hanifi Kokaçya, Esra Doğru, Nihan Katayıfcı, Özden Canbay, Fatma Aman

**Affiliations:** 1 Mustafa Kemal University Faculty of Physical Therapy and Rehabilitation, Hatay, Turkey; 2 Mustafa Kemal University Faculty of Medicine, Department of Psychiatry, Hatay, Turkey

**Keywords:** Child obesity, active video game, exercise, self-esteem, depression

## Abstract

**Objective:**

The aim of this study was to determine the effects of active video games and music-accompanied aerobic and callisthenic exercises on body mass index (BMI), body fat ratio, physical performance tests, psychosocial status, and self-respect in overweight and obese adolescents.

**Methods:**

Fifty (21 males and 29 females) slightly overweight and obese participants with no chronic disorder and of an average age of 12.16±0.99 years were included in the study. The percentile values for BMI, triceps skinfold thickness, waist circumference measurements, and physical performance tests were evaluated. The effects of obesity on psychological wellness were evaluated using the depression scale for children (DSC) and the Piers-Harris Children’s Self-Concept Scale for self-esteem. Following these evaluations, the participants were subjected to an exercise program in five groups of 10 people, 3 days a week for a duration of 8 weeks. Each exercise session lasted 45 minutes. Participants were re-evaluated at the end of the exercise program. The data collected both before and after the exercise program were analyzed using the SPSS 18.0 program.

**Results:**

According to BMI reference values, 28% of the 50 participants (n=14; 6 males and 8 females) were assessed to be overweight and 72% to be obese (n=36; 15 males and 21 females). Following the exercise program, 14% of the participants (n=7; 3 males and 4 females) were assessed as normal, 46% (n=23; 14 males and 9 female) as slightly overweight, and 40% (n=20; 4 male and 16 female) as obese. It was determined that the decrease in BMI values (p<0.05) was higher in male participants than in female participants and that the frequency of obesity was higher in the females. A statistically significant decrease in BMI values was found after the exercise program (p<0.01). Following the exercise program, statistically significant differences have also been observed in the self-esteem (p<0.01), psychological wellness (p=0.025), triceps skinfold thickness, as well as in waist circumference and BMI values of the participants compared to the pre-exercise phase (p<0.01).

**Conclusion:**

An exercise program applied with active video games was found to have positive effects on the obese state as well as on the psychosocial status and self-esteem of obese individuals, indicating that exercise and physical activity have an important role in improvement of the obese state in childhood as well as having positive contributions to self-esteem and psychological wellness state.

**WHAT IS ALREADY KNOWN ON THIS TOPIC?**

Children at risk of overweight have been shown to benefit from increased physical activity administered as a structured program. However, children can get bored of exercise quickly so it is essential to make those exercises interesting.

**WHAT THIS STUDY ADDS?**

After applying an exercise program which was accompanied by active video games, we found significant difference in self-esteem, psychological wellness, performance tests, and body mass index compared to the pre-exercise phase.

## INTRODUCTION

Obesity is a result of an imbalance between nutrient intake and energy expenditure (1). Due to the influence of technology and ever changing social structure, people now spend increasing amounts of their time in front of the television or computer. The risk of obesity increases with a reduction in physical activity. Negative changes in dietary habits combined with a decrease in energy expenditure are the leading causes of this major public health problem. Obesity adversely affects quality of life because of its negative physiological, psychological, systematic, and social effects (2).

Obesity is a condition which leads to various health problems. Its frequency is increasing rapidly amongst adults, children, and adolescents all over the world (3,4). It has been reported that in developed countries, as many as 33% of adults and 20-27% children and adolescents are obese (5). According to the 2010 Turkish Nutrition and Health Research pre-work report, the frequency of obesity in the 15 years and older population is 20.5% among men, 41% among women, and 30.3% in total. The same report states that the frequency of obesity in Turkish children between the ages of 0 and 5 years is 8.5% (10.1% in boys and 6.8% in girls), and the frequency between the ages of 6 and 18 years is 8.2% (9.1% in boys and 7.3% in girls) (6). According to the Turkish Health Research issued by the Turkish Statistical Institute in 2014, the following body mass index (BMI) findings have been reported in the population 15 years and older: 33.7% overweight, 42.2% average weight, and 4.2% underweight. By gender, 24.5% women are obese and 29.3% are overweight, while 15.3% men are obese and 38.3% are overweight (7).

While determination of obesity among adults is calculated by BMI, this may not be appropriate for very young children. Weight and height values for age and gender are also evaluated in assessment of obesity in children. It is also important that local standard curves, if available, are used in this assessment (8).

Although many studies have been conducted to clarify the etiopathogenesis of obesity, all of them have pointed to a multiple etiology (9). Obesity causes various health conditions and chronic illnesses such as cardiovascular disease, hypertension, and diabetes in both adults and children (10,11). Studies have revealed that one third of those who were obese during their childhood and 80% of those who were obese in adolescence were obese as adults. It has also been reported that 30% of obesity cases among adults have a history of childhood obesity (12). Since obesity increases the risk of developing chronic diseases, mortality and morbidity, it is a significant risk factor for children (13).

Furthermore, being overweight and obese affects self-confidence, general appearance, and social activity. A study revealed that depression, lack of self-confidence, and behavior problems were more frequent among clinically obese adolescents (14). It has also been reported that obesity has adverse effects on depression and self-confidence among children (15).

Daily energy need varies with respect to age, gender, occupation, genetic, and physiological characteristics. In order to maintain a healthy weight, the amount of energy that is ingested needs to be balanced to the amount that is spent. Healthy nutrition and adequate physical activity have primary roles in maintaining this balance and in increasing quality of life. Regular physical activity is fundamental for children and adolescents to grow up to be a healthy adult. It is also helpful in avoiding acquisition of bad habits, in being social, and in protection from various chronic diseases (16).

Pharmacotherapy options for the treatment of pediatric obesity are very limited. Therefore, a comprehensive management program that includes exercise and behavior modification is crucial (17). It is essential to make exercise enjoyable and interesting for children because they can get bored of exercise quickly (18). In this study, we hypothesize that active video games and music-accompanied aerobic and calisthenic exercises will contribute positively to children’s BMI percentile and psychosocial status.

## METHODS

This study was implemented within the scope of Supporting Scientists Program of the Scientific and Technological Research Council of Turkey (TÜBİTAK). After obtaining parental consent, primary school students were screened for suitability to participate in our study. Our target number was 50 overweight and obese participants. Evidence of chronic disease was an exclusion criterion.

During the evaluation process, the height and weight of the participants were measured using sensitive scales with sensitivities within 1 cm and 100 grams. BMI values were calculated as weight in kilograms divided by height in meters squared; children with BMI over the 85th percentile were included in the study. The reference percentile values for Turkish children were used in the evaluation of the data (13). Triceps skinfold thickness (in millimeters) measured by means of a caliper (Harpenden Anthropometry) and waist circumference (in centimeters) measured by means of a tape measure were used to determine body fat mass (19). Questionnaires were completed to determine age, gender, obesity history, possible chronic disease history, dietary habits, and problems that they might encounter during their daily life activities using face to face interviews. Physical performance tests were implemented to measure how many seconds it takes them to ascend and descend 20 stairs, the number of squats they can perform in 120 seconds, the amount of time they take to run 50 meters (in seconds), and the number of times they can jump over a rope in 30 seconds. The effects of obesity on psychological wellness were measured using the Depression Scale for Children (DSC) and the effects of obesity on self-esteem were measured using the Piers–Harris Scale (20,21).

### Body Mass Index

In children, BMI may vary with age, rate of growth, and pubertal stage (22,23). At present, gender-specific BMI-for-age percentile charts for the pediatric age group are available in many countries. According to these charts, children and adolescents with a BMI over the 85^th^ but less than the 95^th^ percentile for age and gender are considered as overweight and those with a BMI greater than the 95th percentile as obese (17). The International Obesity Task Force has published an international standard growth chart that enables comparison of prevalence globally (8,17). Nevertheless, many countries prefer to use their own country-specific growth chart because of national features (17). We used reference percentile values for Turkish children (13).

### Waist Circumference

The waist circumference was measured via a tape measure at the umbilicus level (19).

### Triceps Skinfold Thickness

The skinfold thickness can be measured at 10 different points on the body and they are considered as an accurate indication of body fat mass. The subscapular or the left triceps are suggested as the measurement sites in most reports. In this study, the triceps measurement was used and implemented at the exact middle point between acromion and olecranon by using a skinfold caliper, with the arm of the subject hanging vertically at the side (19).

### Depression Scale for Children

DSC was developed by Kovacs (20) in 1992 to measure level of depression in children and adolescents between the ages of 6 and 17 years. This scale consists of 27 articles and evaluates the previous 2 weeks of the child’s life. Each item is scored as 0, 1, or 2 with increasing numbers indicating increased level of depression. The cut-off score is 19 and individuals with scores above this score are accepted as depressed. The adaptation to Turkish validation and credibility were completed by Öy (24) in 1990.

### Piers-Harris Scale

This scale was developed by Piers and Harris in 1964 to measure the self-concept of children between the ages of 9 and 16 years (21). Self-concept refers to the knowledge and perceptions that individuals have of themselves and their behavior. This scale measures the self-concept of children, the development of this concept, and its dimensions and relations with the environment. The answers that the child gives are scored and the child’s self-conception is determined. The adaptation to Turkish validation and credibility was completed by Öner (25) in 1994.

The scale consists of 80 descriptive statements. The answers to those statements are either “Yes” or “No”. The overall score ranges from 0 to 80. The individual’s self-concept is at a more affirmative level as the scores increase. This scale can be completed within 15 to 20 minutes by children with normal development. The scale aims to determine the individual behavior of children and adolescents towards themselves, to study the correlation between these behaviors, and also to evaluate what they feel about themselves.

### Exercise Program

The subjects were divided into five groups of 10 participants each and subjected to an 8-week exercise program for three days per week. The exercise program was as follows:

1) Warm-up exercises: Breathing exercises and slow-paced walking for 10 minutes.

2) Exercise program: Calisthenic and aerobic exercises aimed to target all muscle groups using visual biofeedback with music and active video games for 25 minutes. Three video games that last approximately 6 minutes each were used. Videos were displayed via projection devices and each of the dance moves for the music was demonstrated in the video by an instructor. Between the videos, breathing exercises were implemented combined with slow-paced walking with marked times.

3) Cool-down exercise: Mild stretching, slow-paced walking, and breathing exercises for 10 minutes. The program was completed with a relaxation posture.

The video games were transferred to CDs and distributed to the participants at the end of the 8-week training period in order to maintain the continuity of the exercises and enable them to acquire regular exercising habits.

The data acquired before and after the exercise program were evaluated using the SPSS 18.0 program (SPSS Inc., Chicago, IL, USA). The relationship between categorical variables was evaluated using the chi-square test, the relationship between gender and the continuous variables was evaluated using the Mann-Whitney U test, and the comparison of the data before and after the exercise program was evaluated using the Wilcoxon signed-rank test. A p-value <0.05 was considered statistically significant.

## RESULTS

We screened 80 adolescents for participation in the study and 50 participants met all the eligibility requirements and were included in the study (Table 1).

The dietary habits, daily meal times, and family obesity history of the participants were examined. It was determined that 56% of the study subjects were receiving a balanced diet and the remaining 44% did not. Number of meals per day was only 2 in 10% of the participants, 64% had 3 meals per day, 24% ate 4 meals per day, and 2% ate 5 or more times per day. Examination of family history revealed that 42% of the participants had a first-degree relative with obesity.

We determined that 14 (28%) of the participants were overweight and 36 (72%) were obese before the exercise program. After the exercise program, the BMI values of the subjects had decreased significantly (p<0.05) (Table 2).

Triceps skinfold thickness measurement values as well as waist circumference measurements also showed a decrease after the exercise program (p<0.05) (Table 3).

The 50 meter run time, the number of squats in 120 seconds (s), the time to ascend and descend 20 stairs, and the number of times jumping over a rope in 30 seconds had all improved after the exercise program (p<0.05) (Table 4).

Before the exercise program, 18 participants exhibited depressive findings, whereas after the exercise program, the number had decreased to 13. We determined that children demonstrated affirmative development in their psychological wellness after the exercise program (p<0.05) (Table 5).

A significant difference was recorded in the Piers-Harris Self-Respect sub-components of behavior, intelligence, school status, popularity, happiness, physical appearance, and anxiety levels after the exercise program (p<0.05) (Table 6).

In the evaluation form that we drew up to evaluate their dietary habits, the participants were asked whether they had a balanced nutrition, meaning eating natural homemade foods at regular times, and how many meals they had daily. Twenty-eight out of 50 participants (56%) answered “yes” when asked whether they had a balanced nutrition.

Upon evaluating the answers to these questions gender-wise, no statistical differences were recorded between the answers by the male and female participants.

## DISCUSSION

Improved diet, increased exercise, and change in eating habits as well as involving the family in the treatment process are essential to prevent the continuation of obesity. The addition of behavior treatment to the exercise and diet treatment already used to correct childhood/adolescent weight problems has proven to produce better results (3).

Since obese people are inclined to move less, exercise should not be neglected during their treatment. The purpose of exercise for obese people is not only to lose weight but also help them acquire a behavioral change for a healthier life style (26). For improvement of the obese state, it is essential that all adolescents exercise daily and accept it as part of their lives. Through exercise, the loss of fat increases and lean tissue mass is preserved (5).

By means of an 8-week exercise program, a highly significant decrease in triceps skinfold thickness and waist circumference measurements were observed in the participants of this study. These results may have long-term implications for our participants. Lack of physical activity leads uninformed adolescent and obese adults into a sedentary life which will increase the risk of cardiovascular, pulmonary, and metabolic diseases. Furthermore, obesity itself can cause chronic complications such as hypertension, stroke, cardiac diseases, thrombogenesis, pulmonary diseases, endometrial and colon cancers, sleep apnea, dyslipidemia, gallbladder stone, type 2 diabetes, gout and pain, or dysfunction in the locomotor system. These complications lead to a decrease in the life expectancy of the obese individual (15). Early intervention, such as that made in our study, may improve the quality of life of these individuals immediately, as shown by the changes in their self-esteem and depression scores.

In our study, 36% of the participants were found to be depressed. Their self-esteem was also observed to be low. These results agree with a study by Pınar (27) that examined the level of depression and self-esteem in 87 obese women. The author reported that 42.5% of the obese participants were depressed and 58.6% of them had low self-esteem. In addition, in a study by Kartal (28), it was demonstrated that obese individuals had a lower level of self-esteem. Following the exercise program, our participants demonstrated a positive development in their psychological wellness (p=0.025). The number of our participants with depressive symptoms decreased from 36% to 26% after the exercise program. Moreover, compared to the pre-exercise program, a statistically significant improvement was recorded in the total Piers-Harris Self-Esteem points and their sub-components such as behavior, intelligence, education, popularity, happiness, physical appearance, and anxiety (p=0.000).

The effects of physical activity on obesity vary with age. For morbid obesity which may be accompanied by diabetes, cardiac disease, hypercholesterolemia, and arthritic diseases, low intensity exercises are suggested (25% VO_2_ max). The exercises help burn esterified fatty acids. For obese people, exercises that do not involve a strain on body weight such as swimming, cycling, and mat exercises (exercises on the mattress) should be chosen. Ergometric exercises result in 10-12% fat loss. All the training programs should be rhythmic exercises that do not push the respiratory and circulatory systems too far. For other obese individuals, mildly intense exercises are advised (65% VO_2_ max). These exercises stimulate the breakdown of intramuscular triglycerides. In addition, walking, dancing, treadmill use, water sports, gardening, and house chores are recommended (29).

In a randomized, controlled study which Maddison et al (30) carried out in 322 children (aged 10-14 years) in New Zealand, they applied 60 minutes of mildly intense physical activity during which the study group was exposed to active video games and the control group was exposed to non-active video games. They determined that the active video games had positive effects on the body composition and there was a significant increase in vital capacity.

In another study involving sedentary adolescents in New York, a comparison was made between a group that was exposed to a 30-minute active video game and another group that was given a 30 minute treadmill walk. It was observed that the video game group had a significant increase in their heart rate and pulse in comparison to the treadmill group, showing that active video games can be an alternative to aerobic exercises when it comes to physical activity (31).

Barbeau et al (32) demonstrated that a total of 80-minute activity program that consisted of 35 minutes aerobics, 20 minutes stretching, and 25 minutes skills development and which was offered every day during the school year, had significant effects on BMI and led to a decrease in body fat.

In this present study, an exercise program consisting of 10 minutes warm-up, 25 minutes aerobics exercise, and 10 minutes cool-down, which totaled 45 minutes and was accompanied by active video, was applied to participants for three days each week for 8 weeks. The exercise program resulted in significant decreases in the BMI percentile values of participants (p=0.022). Furthermore, we found that male participants had greater changes than female participants (Table 5). Generally in these age groups, boys are more interested in video or computer games than girls and this might be the reason for this result. In addition, physical performance tests revealed that there were significant improvements in the running time, stair climbing, number of squats and rope jumps compared to the pre-program findings (p=0.00).

In conclusion, the results of this study showed that the exercise program accompanied by active video that we applied had positive effects on the obese state as well as on the psychosocial status and self-esteem of our participants. In this study, exercises were made enjoyable for the participants, which caused them to become more active and willing. This finding was noted especially in the boys. We know that physical activity has a major role in preventing childhood obesity and can lead to a healthy and active lifestyle. Our study showed that active video game participation in exercise is effective for the improvement of the obese state and possibly for prevention of obesity in children.

## Ethics

Ethics Committee Approval: The study protocol was approved by the Ethics Committee of Mustafa Kemal University Faculty of Medicine at 13.01.2014, Informed Consent: Informed consent was obtained from all parents.

Peer-review: Externally peer-reviewed.

## Figures and Tables

**Table 1:**
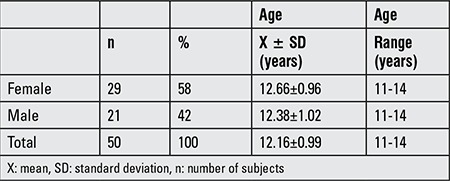
The demographic characteristics of the participants (n=50)

**Table 2:**
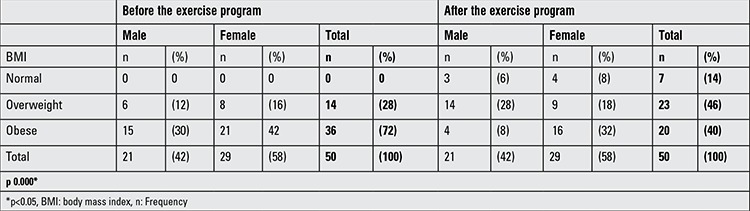
Classification of the participants based on body mass index before and after the exercise program

**Table 3:**
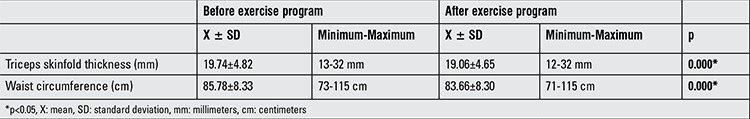
Triceps fat mass and waist circumference measurements after the exercise program

**Table 4:**
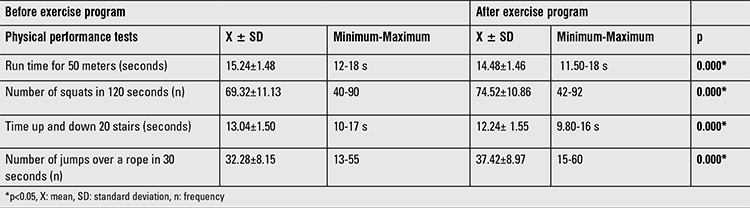
Physical performance tests before and after the exercise program

**Table 5:**
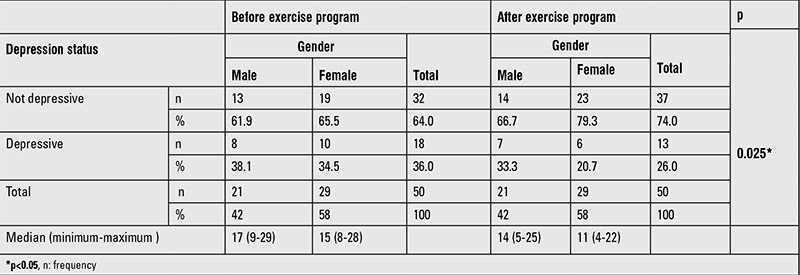
Depression status before and after the exercise program

**Table 6:**
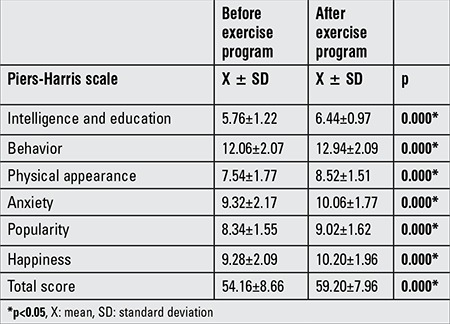
Piers-Harris Self-Respect Scale scores before and after the exercise program
